# Neuromyelitis optica pathology in rats following intraperitoneal injection of NMO-IgG and intracerebral needle injury

**DOI:** 10.1186/2051-5960-2-48

**Published:** 2014-04-24

**Authors:** Nithi Asavapanumas, AS Verkman

**Affiliations:** 1 Departments of Medicine and Physiology, University of California, San Francisco, CA, USA

**Keywords:** NMO, Aquaporin-4, Astrocyte, Complement, Neuroinflammation

## Abstract

**Introduction:**

Animal models of neuromyelitis optica (NMO) are needed for drug testing and evaluation of NMO disease pathogenesis mechanisms.

**Results:**

We describe a novel passive-transfer model of NMO in which rats made seropositive for human anti-aquaporin-4 (AQP4) immunoglobulin G antibody (NMO-IgG) by intraperitoneal (IP) injections were subject to intracerebral needle injury. Following a single IP injection, NMO-IgG distributed rapidly to peripheral AQP4-expressing cells (kidney collecting duct, gastric glands, airways, skeletal muscle) and area postrema in brain, but not elsewhere in the central nervous system; however, no pathology was seen in brain, spinal cord, optic nerve or peripheral tissues. After testing various maneuvers to produce NMO-IgG-dependent pathology in brain, we found that transient puncture of brain parenchyma with a 28-gauge needle in NMO-IgG seropositive rats produced robust NMO pathology around the needle track, with loss of AQP4 and glial fibrillary acidic protein, granulocyte and macrophage infiltration, centrovascular deposition of activated complement, and blood–brain barrier disruption, with demyelination by 5 days. Pathology was not seen in rats receiving control (non-NMO) human IgG or in NMO-IgG-seropositive rats made complement-deficient by cobra venom factor. Interestingly, at 1 day a reversible, multifocal astrocytopathy was seen with loss of AQP4 and GFAP (but not myelin) in areas away from the needle track.

**Conclusions:**

NMO-IgG-seropositivity alone is not sufficient to cause NMO pathology in rats, but a single intracerebral needle insertion, without pre-existing inflammation or infusion of pro-inflammatory factors, was sufficient to produce robust NMO pathology in seropositive rats.

## Introduction

Neuromyelitis optica (NMO) is an inflammatory demyelinating disease of the central nervous system that can produce motor and visual impairment [1-3]. Most NMO patients are seropositive for immunoglobulin G autoantibodies (NMO-IgG) directed against aquaporin-4 (AQP4) [4,5], a water channel expressed in the plasma membrane of astrocytes in brain, spinal cord and optic nerve [6,7]. Though AQP4 is also expressed in some peripheral tissues, including kidney collecting duct, gastric glands, airway epithelia and skeletal muscle [8,9], significant pathology is absent in peripheral tissues in NMO [3]. NMO lesions in the human central nervous system show astrocyte damage with loss of AQP4 and glial fibrillary acidic protein (GFAP), inflammation with granulocyte and macrophage infiltration, vasculocentric deposition of activated complement, blood–brain barrier disruption and demyelination [4,5,10-12]. There is a substantial body of evidence supporting a pathogenesis mechanism in which NMO-IgG binding to astrocytic AQP4 produces complement-dependent cytotoxicity (CDC), which leads to inflammation and blood–brain barrier disruption with secondary oligodendrocyte injury, demyelination and neuronal injury [13-15]. Antibody-dependent cellular cytotoxicity (ADCC) also plays a role [16], as does, perhaps, AQP4-sensitized T cells or other factors promoting blood–brain barrier breakdown [17-19].

There is considerable interest in creating animal models of NMO for investigation of disease pathogenesis mechanisms and testing therapeutics [20,21]. The original animal models involved intraperitoneal injection of IgG purified from NMO patient serum in rats with pre-existing inflammation produced by sensitization to myelin oligodendrocyte protein (experimental autoimmune encephalomyelitis, EAE) [22-24] or by complete Freund’s adjuvant [25]. In these models greater CNS inflammation was seen in rats receiving NMO-IgG, with evidence for astrocyte damage and complement activation. However, the pre-existing inflammation in these models confounds data interpretation because NMO involves astrocyte-targeted antibodies rather than sensitized T cells.

Mouse models of NMO involving intracerebral injection or infusion of NMO-IgG and human complement have been informative in studying disease pathogenesis mechanisms, such as the roles of ADCC [26,16] and of various leukocyte types [27-29]. Brain pathology in injected mice is similar to NMO pathology in humans, with loss of AQP4, GFAP and myelin, granulocyte and macrophage infiltration, and complement deposition [19]. In recent advances, including the use of NMO superantibodies with increased CDC/ADCC effector function(s) and CD59 knockout mice, optic neuritis [30] and longitudinally extensive transverse myelitis [31] have been produced in mice by passive transfer of NMO-IgG and human complement. However, all mouse models require direct administration of human complement into the central nervous system, as the mouse complement system is ineffective because, in part, of circulating complement-inactivating protein(s) [32].

To overcome the limitations of existing models, and building on methods developed in mice, we recently reported a rat model of NMO involving intracerebral injection of NMO-IgG, without complement supplementation and without pre-existing neuroinflammation [33]. Unlike mice, rats have an active complement system similar to humans. The NMO-IgG injected rats developed characteristic NMO pathology around the needle track, which was complement-dependent. The model was applied to investigate the role of ADCC and macrophages in NMO pathogenesis.

A limitation of our reported rat model [33] was the need to inject NMO-IgG directly into the brain, which is different from human NMO in which NMO-IgG is present in serum and pathology is initiated, in large part, following NMO-IgG entry into the central nervous system. Testing of certain therapeutics, such as aquaporumab antibodies [34], IgG inactivation therapies [35,36] and complement-targeted drugs [37,38], are best done in models of NMO in which pathology is produced in seropositive animals. Motivated by this need, the goal of this study was to establish a robust of NMO in rats made NMO-IgG-seropositive by peripheral NMO-IgG administration. We first studied the tissue distribution and serum pharmacokinetics of peripherally administered NMO-IgG in rats, and then established the minimal conditions in which robust NMO pathology could be produced in seropositive rats.

## Materials and methods

### Rats

Lewis rats were purchased from Charles River Lab (Wilmington, MA). Experiments were done using weight-matched rats (150–250 g), age 8 to 12-weeks. Protocols were approved by the University of California San Francisco Committee on Animal Research.

### Antibodies and sera

A recombinant monoclonal NMO antibody, rAb-53 (referred to as NMO-IgG), was generated from a clonally expanded plasma blast population from cerebrospinal fluid of an NMO patient, as described and characterized previously [22,39]. NMO serum was obtained from seropositive individuals who met the revised diagnostic criteria for clinical disease [3]. Non-NMO (seronegative) human serum was used as control. In some studies IgG was purified from NMO or control serum using Protein A-resin (GenScript, Piscataway, NY) and concentrated using Amicon Ultra Centrifugal Filter Units (Millipore, Billerica, MA).

### Cell culture and cytotoxicity assay

Chinese hamster ovary (CHO) cells stably expressing human M23-AQP4 [40] were cultured at 37°C in 5% CO_2_ 95% air in F-12 Ham’s Nutrient Mixture medium supplemented with 10% fetal bovine serum, 200 μg/ml geneticin (selection marker), 100 U/ml penicillin and 100 μg/ml streptomycin. For assay of complement-dependent cytotoxicity (CDC) in seropositive rats, cells were plated on 96-well microplates, washed with phosphate-buffered saline (PBS) and incubated at 28°C for 60 min with different concentration of heat-inactivated rat serum and 5% human complement (Innovative Research, Novi, MI) in a total volume of 50 μl. Cytotoxicity was measured by the Alamar Blue assay (Invitrogen). In some experiment, the activity of rat complement was measured by incubating 5% rat serum plus 10 μg NMO-IgG in M23-AQP4 expressing CHO cells.

### Pharmacokinetics and tissue distribution

Adult rats received 750 μg of NMO-IgG (or control IgG) in PBS by intraperitioneal injection in a total volume of 500 μl. Blood was collected through the tail vein at 1, 2, 4, 6, 8, 24 and 48 h, left for 30 min at room temperature to allow clotting, and centrifuged for 10 min at 3000 g, 4°C. Serum was diluted 100-fold and human IgG concentration was determined using a human IgG ELISA kit (GenWay, San Diego, CA). For analysis of tissue distribution, at 24 h after injection rats were anesthetized using ketamine (75–100 mg/kg) and xylazine (5–10 mg/kg) and perfused with PBS and then PBS containing 4% paraformaldehyde (PFA). AQP4-expressing tissues were removed, post-fixed overnight in 4% PFA and dehydrated overnight in 30% sucrose. Tissues were embedding in OCT compound (Sakura Finetek, Torrance, CA) for sectioning and immunostaining.

### NMO-IgG delivery and intracerebral needle injury

Adult rats were administered 1 mg NMO-IgG (or control IgG) in a volume of 500 μl 6 h before and 24 h after intracerebral needle injury. To create the needle injury, rats were anesthetized with intraperitoneal ketamine (75–100 mg/kg) and xylazine (5–10 mg/kg) and mounted in a stereotaxic frame. Following a midline scalp incision, a burr hole of diameter 1 mm was made in the skull 3.5 mm to the right of the bregma. A 28-gauge needle attached to 10-μl gas-tight glass syringe (Hamilton, Reno, NV) was inserted 5 mm deep to infuse 10 μl of PBS (at 2 μl/min). After 1 or 5 days, rats were anesthetized and perfused through the left cardiac ventricle with 100 ml PBS and then 25 ml of PBS containing 4% PFA. In some studies rat complement was depleted by intraperitoneal injection of cobra venom factor (350 U/kg; Quidel Corporation, Santa Clara, CA) [41,42] 24 h before and 48 h after intracerebral needle injury.

### Immunofluorescence

Five micrometer-thick paraffin sections were immunostained at room temperature for 1 h with antibodies against rat AQP4 (1:200, Santa Cruz Biotechnology), GFAP (1:100, Millipore), myelin basic protein (MBP) (1:200, Santa Cruz Biotechnology), ionized calcium-binding adaptor molecule-1 (Iba1; 1:1,000; Wako), albumin (1:200, Santa Cruz Biotechnology), Ly-6G (1:100, Santa Cruz Biotechnology), C5b-9 (1:50, Hycult Biotech), CD45 (1:10, BD Biosciences), CD163 (1:50, Bio-Rad Laboratories), neurofilament (1:200, Millipore), iNOS (1:100, BD Biosciences) or arginase-1 (1:50, Santa Cruz Biotechnology), followed by appropriate fluorescent secondary antibody (1:200, Invitrogen) or biotinylated secondary antibody (1:500, Vector Laboratories). Tissue sections were examined with a Leica (Wetzlar, Germany) DM 4000 B microscope. AQP4, GFAP and MBP immunonegative areas were defined by hand and quantified using ImageJ. Data are presented as area (mm^2^) of immunonegative area.

### Statistical analysis

Comparisons between two groups were performed using an unpaired *t*-test. P < 0.05 was considered statistically significant. Values are presented as mean ± S.E.

## Results

### NMO-IgG pharmacokinetics and tissue distribution following IP injection

**Figure 1:**
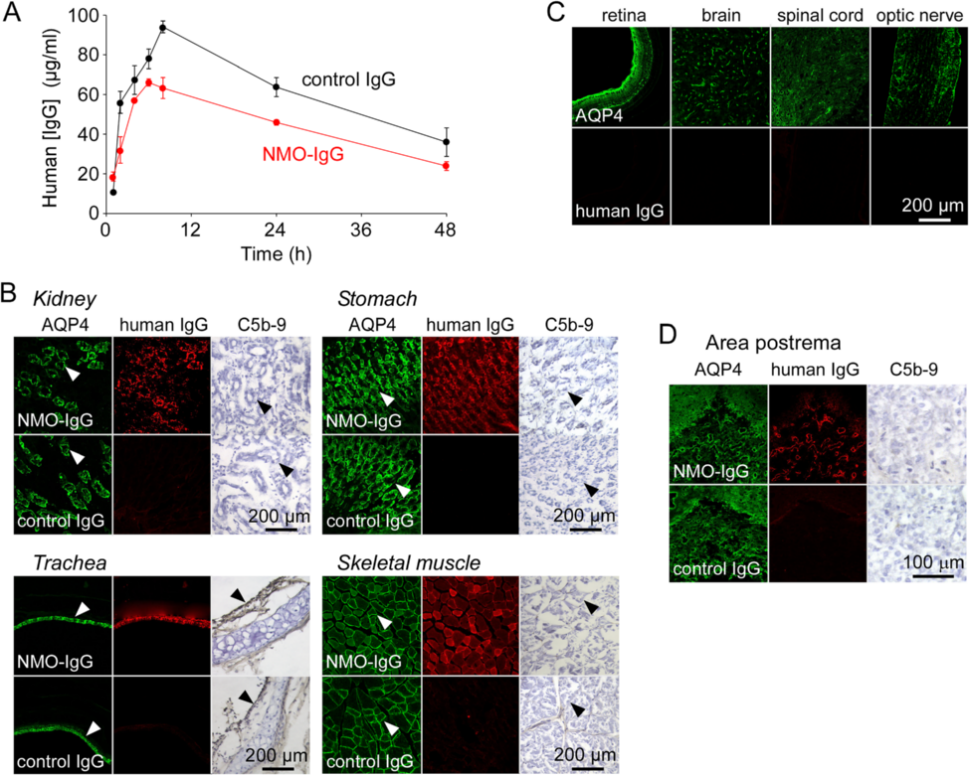
**Pharmacokinetics and tissue distribution of NMO-IgG following IP injection. A**. Serum concentration of human IgG quantified by ELISA at 1, 2, 4, 6, 8, 24 and 48 h after intraperitoneal injection of NMO-IgG or control IgG (each 750 μg) (S.E., 3 rats per group). **B**. Tissue distribution of NMO-IgG at 24 h after injection of NMO-IgG or control IgG. AQP4 immunofluorescence (green), human IgG (with secondary anti-human antibody, red) and activated complement (C5b-9). Arrows indicate basolateral membrane in kidney inner medullary collecting duct epithelial cells, basolateral membrane of gastric parietal cells, basolateral membrane of tracheal surface epithelial cells, and the plasmalemma in skeletal muscle. **C**. AQP4 and human IgG staining in retina, brain, spinal cord and optic nerve in same rats as in **B**. **D**. AQP4, human IgG and C5b-9 staining in area postrema in same rats as in **B**.

The pharmacokinetics of NMO-IgG in rats was determined following a single intraperitoneal injection of 750 μg of NMO-IgG (recombinant human monoclonal antibody rAb-53) or control (non-NMO) human IgG. Human IgG concentration in rat serum was assayed by ELISA against human IgG, which is not sensitive to rat IgG. Figure 1A shows human IgG concentration in rat serum over 48 h. NMO-IgG concentration increased over the first few hours as it was absorbed from the peritoneal cavity, was maximum at ~6 h, and then decreased with t_1/2_ ~ 48 h. The higher levels seen for an equivalent amount of control (non-NMO) IgG are probably related to binding of NMO-IgG in AQP4-expressing tissues.

The tissue distribution of injected NMO-IgG was determined by immunofluorescence in which fixed tissues were immunostained for AQP4 using an anti-AQP4 antibody and for NMO-IgG using an anti-human fluorescent secondary antibody. Figure 1B shows colocalization of NMO-IgG and AQP4 in the basolateral membrane of kidney inner medullary collecting duct epithelial cells, the basolateral membrane of gastric parietal cells, the basolateral membrane of tracheal surface epithelial cells, and the plasmalemma in skeletal muscle. Immunofluorescence with the anti-human secondary antibody was negative in rats receiving control IgG. C5b-9 immunofluorescence was negative in each tissue, indicating absence of deposition of activated complement. A similar pattern, though with less intense NMO-IgG staining, was seen at 2 h after injection (not shown).

Figure 1C shows absence of NMO-IgG deposition at 24 h in retina, brain, spinal cord and optic nerve – CNS tissues in which AQP4 is expressed in retinal Muller cells, and astrocytes in brain, spinal cord and optic nerve. By an extensive search of sections from these tissues we found NMO-IgG in circumventricular organs, including area postrema (Figure 1D), which lack an intact blood brain barrier. However, deposition of activated complement was not seen (Figure 1D), nor was there evidence of NMO pathology with loss of AQP4, GFAP or myelin (not shown).

### NMO model in NMO-IgG seropositive rats

**Figure 2:**
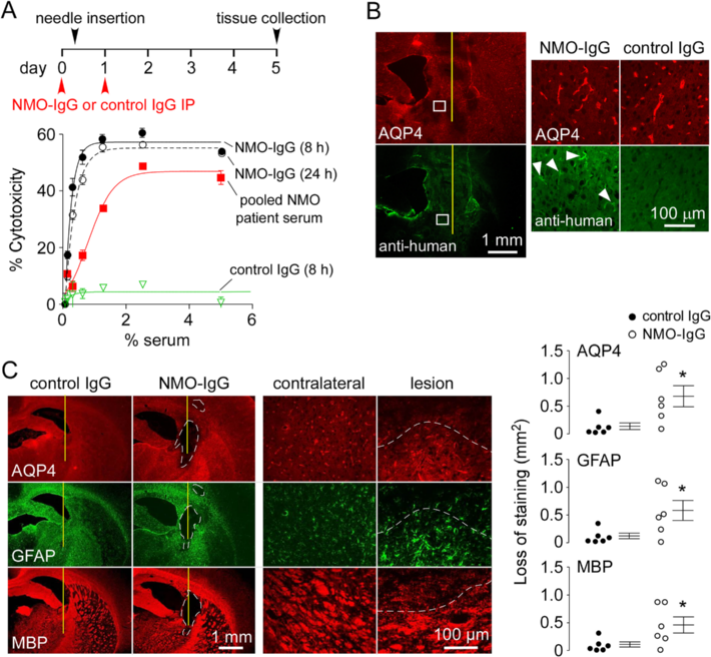
**NMO pathology in seropositive rats at 5 days after intracerebral needle injury. A**. (top) NMO-IgG administration and intracerebral needle injury protocol. (bottom) Rats received 1 mg NMO-IgG or control IgG by intraperitoneal injection. Cytotoxicity in rat serum obtained at 8 and 24 h was measured by Alamar blue assay in AQP4-expressing CHO cells at 1 h after incubation with different concentration of heat-inactivated rat serum and 5% human complement (S.E., n = 3). For comparison data shown for heat-inactivated pooled human sera from NMO seropositive patients to which 5% human complement was added. **B**. (left) AQP4 and human IgG staining in brain of rats receiving NMO-IgG at 1 day after intracerebral needle injury. Needle track shown as yellow line. (right) Higher magnification of boxed region along with data for control IgG. **C**. (left) AQP4, GFAP and MBP immunofluorescence at 5 days. Needle track shown as yellow line and lesion demarcated by white dashed line. (middle) Higher magnification. (right) Summary the areas of loss of AQP4, GFAP and MBP immunostaining. Each point is from a different rat.

Because neither NMO-IgG deposition nor NMO pathology was seen in the initial studies, several approaches were tested to produce NMO pathology in rats. An NMO-IgG administration protocol was first established to produce sustained seropositivity in rats. Rats were administered NMO-IgG (or control IgG) (1 mg) by intraperitoneal injection at days 0 and 1 (Figure 2A, top). To confirm seropositivity, rat serum obtained at 8 and 24 h was biossayed for CDC in vitro in which different concentrations of rat serum, after heat inactivation (to inactivate rat complement), were incubated with AQP4-expressing CHO cells in the presence of 5% human complement. Figure 2A (bottom) shows strong seropositivity in the NMO-IgG-treated rats, with greater cytotoxicity in rat serum at 8 and 24 h than that produced by (heat-inactivated) pooled human sera from seropositive NMO patients in the presence of added 5% human complement. No cytotoxicity was seen in serum from rats injected with control (non-NMO) IgG.

After evaluating different approaches to produce robust NMO pathology in NMO-IgG seropositive rats, including stab injury by a surgical blade, osmotic pump delivery, cortical freeze injury and others, we found that a single intracerebral needle injury produced NMO pathology after 5 days. A 5-day protocol (Figure 2A, top) was used in which an intracerebral needle injury (5-min insertion of a 28-gauge needle with infusion of 10 μl PBS) was created 6 h after intraperitoneal NMO-IgG administration, and followed 18 h later by a second NMO-IgG administration. Figure 2B shows that NMO-IgG entered rat brain parenchyma at 1 day following the intracerebral needle injury in this model, with localization near the needle track and in some structures away from the needle track. At high magnification the distribution of NMO-IgG overlapped with that of AQP4, which was concentrated at astrocyte end-feet around capillaries. No AQP4 binding was seen in rats receiving control IgG.

Pathology with characteristic features of NMO was seen in rats at 5 days after NMO-IgG administration, with loss of AQP4, GFAP and myelin (MBP) immunofluorescence around the needle track (Figure 2C, left). At high magnification the area of AQP4, GFAP and MBP loss was well-demarcated. Pathology was not seen in rats administered control IgG. Figure 2C (right) summarizes the areas of loss of staining.

### Characterization of rat NMO model

**Figure 3:**
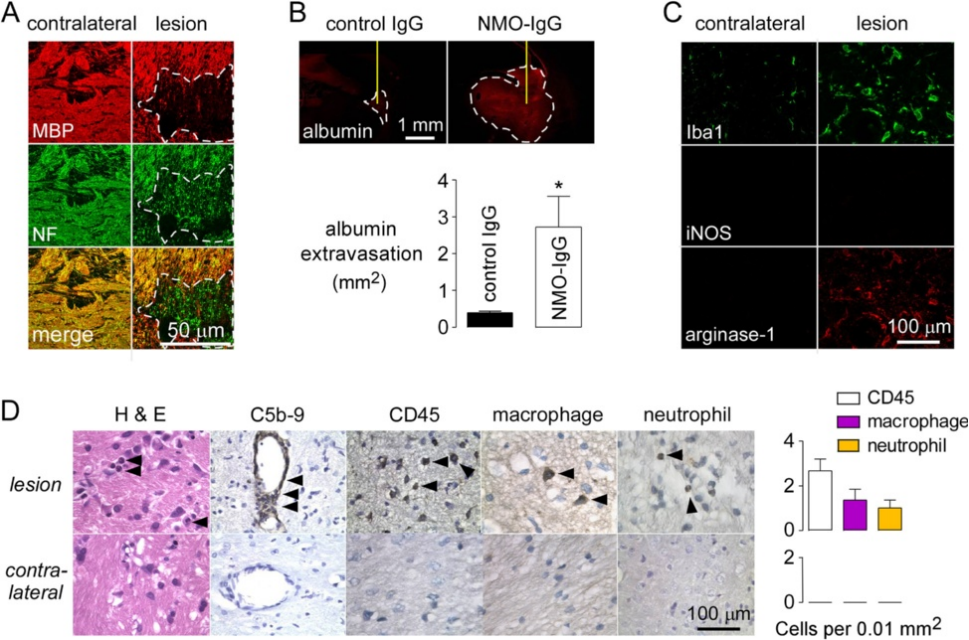
**Inflammation and blood–brain barrier disruption in the NMO rat model. A**. MBP and neurofilament (NF) immunofluorescence at 5 days in rats treated as in Figure 2. White line demarcates the lesion. **B**. Albumin immunofluorescence shown with quantification of immunopositive area (S.E., n = 3). **C**. Iba1, iNOS (M1-macrophage marker) and arginase-1 (M2-macrophage marker) immunofluorescence. **D**. (left) Hematoxylin and eosin (H&E) staining, and immunofluorescence for activated complement (C5b-9), leukocytes (CD45), neutrophils (Ly6-G) and macrophages (CD163) in brain at 5 days. (right) Summary of the number of infiltrating cells per 0.01 mm^2^ (S.E., 3 rats).

In addition to loss of AQP4 and GFAP, other characteristic features of NMO pathology include demyelination with axon preservation, blood–brain barrier disruption, microglial activation, centrovascular deposition of activated complement, and granulocyte and macrophage infiltration. Figure 3A shows a typical demylinating lesion at 5 days as seen by loss of MBP immunofluorescence but preservation of axons as seen by neurofilament immunofluorescence. Substantial blood–brain barrier disruption was seen in the NMO-IgG treated rats as seen by albumin extravasation around the needle track (Figure 3B). Iba1 immunofluorescence showed activated microglia as well as macrophage infiltration in the NMO-IgG treated rats (Figure 3C). Most macrophages were positive for arginase-1 but not iNOS, suggesting predominantly M2-type macrophages at 5 days.

Figure 3D (left) shows H&E staining and immunocytochemistry for activated complement and leukocyte markers. The lesion (with reduced AQP4 and GFAP staining) showed inflammation on H&E staining, vasculocentric deposition of activated complement (C5b-9), and leukocyte infiltration (CD45), consisting mainly of neutrophils (Ly-6G) and macrophages (CD163). The contralateral hemisphere (and rats receiving control antibody, not shown) were negative for each of these markers. Figure 3D (right) summarizes the number of CD45-positive cells per 0.01 mm^2^ in lesions, which contained more macrophages than neutrophils.

**Figure 4:**
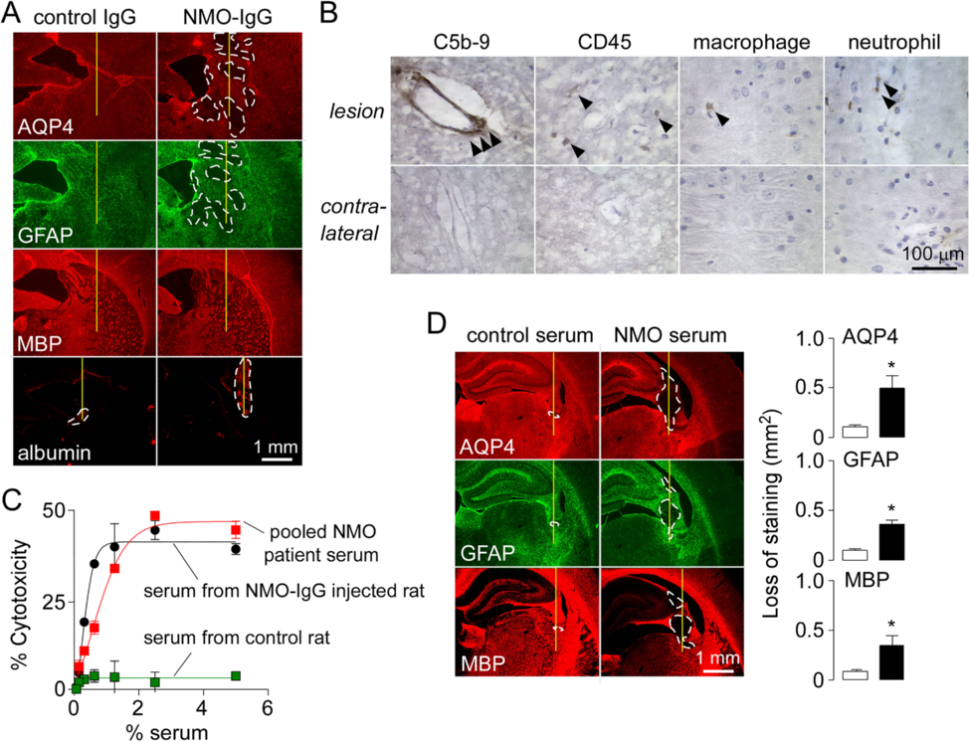
**Early NMO pathology and NMO pathology produce from NMO patient serum. A**. AQP4, GFAP, MBP and albumin immunofluorescence in rat brain at 1 day after intraperitoneal injection of NMO-IgG and intracerebral needle injury. **B**. Staining for activated complement (C5b-9), leukocytes (CD45), neutrophils (Ly6-G) and macrophages (CD163) at 1 day. **C**. Rats were administered 75 mg of purified IgG from NMO patient serum (or non-NMO control serum) as in Figure 2A (top). In vitro cytotoxicity of rat serum obtained at 8 h measured in AQP4-expressing CHO cells by Alamar blue assay as in Figure 2A (bottom) (S.E., n = 3). **D**. (Left) AQP4, GFAP and MBP immunofluorescence at 5 days in rats administered purified IgG from NMO patients (or control non-NMO IgG). (right) Summary the areas loss of AQP4, GFAP and MBP (S.E., 4 rats).

To study the early development of pathology in this model, rats receiving NMO-IgG or control antibody were sacrificed at 1 day after needle injury. Figure 4A shows early astrocyte damage seen as loss of AQP4 and GFAP immunofluorescence, but without myelin loss. Interestingly, multiple small lesions were seen in the general vicinity of the needle track at 1 day, though albumin extravasation was confined to the area directly around the needle track. Pathology was not seen in rats receiving control IgG. Figure 4B shows deposition of activated complement at 1 day with little infiltration of inflammatory cells. The presence of multiple areas of loss of AQP4 and GFAP at 1 day away from the needle track suggests a reversible astrocytopathy that does not progress, perhaps because inflammatory cells have limited access to the more distant nascent lesions.

The characteristic NMO pathology produced by the recombinant NMO-IgG was also seen following intraperitoneal injection of IgG purified from NMO patient serum. The amount of injected purified IgG was chosen to give a similar concentration as in human serum. In vivo CDC produced by serum of rats injected with IgG purified from NMO patient was greater than that produced by pooled human sera from seropositive NMO patients (Figure 4C). Cytotoxicity was not seen in rats injected with control (non-NMO) IgG. Figure 4D shows significant loss of AQP4, GFAP and MBP immunofluorescence at 5 days after two injections of 75 mg IgG purified from NMO patient serum, which was not seen in rats receiving the same amount of IgG from control (non-NMO) human serum.

### NMO pathology in seropositive rats is complement-dependent

**Figure 5:**
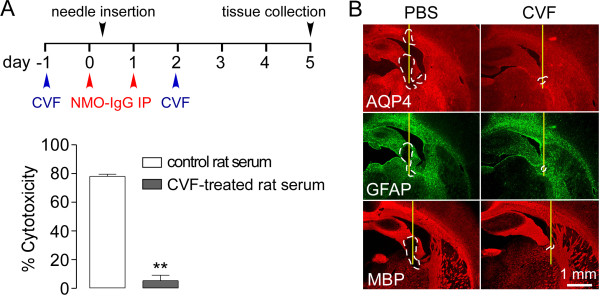
**NMO pathology in seropositive rats is complement-dependent. A**. (top) Experimental protocol. (bottom) Cytotoxicity in AQP4-expressing CHO cells incubated with 10 μg/ml NMO-IgG and 5% serum from cobra venom factor (and control saline) treated rats (S.E., n = 3, ** P < 0.01). **B**. AQP4, GFAP and MBP immunofluorescence at 5 days.

To confirm the involvement of complement in producing NMO pathology our model, rat complement was inactivated with cobra venom factor, a well-established approach to study the involvement of complement in various disease processes [41,42]. Rats received cobra venom factor by intraperitoneal injection 1 day before and 2 days after intraperitoneal IgG delivery, as diagrammed (Figure 5A, top). Figure 5A (bottom) confirmed the loss of complement activity in rat serum in this protocol, as shown using an in vitro CDC assay in which AQP4-expressing CHO cells were incubated with NMO-IgG and rat serum. Figure 5B shows absence of NMO pathology in the cobra venom factor-treated seropositive rats, while robust pathology was seen in control (saline) treated seropositive rats, supporting the requirement for active rat complement in this model.

## Discussion

The goal of this work was to develop a minimally intrusive, robust model of NMO in NMO-IgG seropositive rats. Pharmacokinetic analysis showed that human IgG was effectively absorbed in rats following IP injection, and that the t_1/2_ for NMO-IgG disappearance in blood was ~48 hours. A convenient NMO-IgG dosing regimen was established to maintain rat seropositivity at a level at least as high as that in typical seropositive NMO patients, as verified by a serum cytotoxicity bioassay. Though intravenous NMO-IgG administration could also be used to maintain seropositivity in rats, IP administration is technically easier, particularly when many rats are studied and more than one injection per rat is needed. NMO-IgG administration by mini-pump, though not tested, might also be suitable if subcutaneously delivered antibody is efficiently absorbed.

Tissue distribution studies showed rapid binding of IP-administered NMO-IgG to peripheral AQP4-expressing cells in kidney, stomach, trachea and skeletal muscle, as was found previously in NMO-IgG-injected mice [43]. NMO-IgG was also seen in AQP4-expressing cells in the area postrema of brain, which lacks a blood–brain barrier, but not elsewhere in the central nervous system including spinal cord and optic nerve. Remarkably, though serum complement activity in rats is comparable to that in humans, no pathology was seen in peripheral AQP4-expressing organs or in circumventricular organs, such as area postrema in brain. The reason(s) why peripheral organs are spared in NMO are not clear; it has been speculated that the specialized environment in central nervous system tissues may be responsible, as might the differential expression of complement inhibitor proteins, such as CD55 and CD59, in central nervous system versus peripheral tissues [44-47]. The absence of pathology in circumventricular organs in brains of seropositive rats suggests that the initiation of NMO pathogenesis requires some additional insult, perhaps local inflammation. Of note, humans may be seropositive for many years prior to clinical signs of NMO [48], and an inflammatory disease, such as gastroenteritis, is anecdotally reported to precede clinical NMO disease.

After evaluating several maneuvers to produce NMO pathology in seropositive rats, we found that a single needle insertion into brain parenchyma was sufficient to produce robust lesions around the needle track, with the characteristic pathological features of human NMO including loss of AQP4, GFAP and myelin, vasculocentric deposition of activated complement, granulocyte and macrophage infiltration, and blood–brain barrier disruption. Significant pathology was absent in rats administered non-NMO human IgG. The absence of significant pathology in similarly treated rats receiving NMO-IgG but made complement-deficient by cobra venom factor indicates the requirement for complement in this model. It should be informative to evaluate the roles of ADCC, neutrophils, eosinophils and macrophages in this model, as done previously in other NMO models [26,16-29,33], as well as to test therapeutics targeting complement [37,38], NMO-IgG pathogenicity [35,36], NMO-IgG binding to AQP4 [34,49], and leukocyte-targeted drugs such as sivelestat [27] and cetirizine [29].

The NMO pathology seen at 5 days after intracerebral needle injury in seropositive rats was around the needle track in an area corresponding to that of early NMO-IgG diffusion. At one day astrocyte damage with loss of AQP4 and GFAP was seen, but little myelin loss. Interestingly, at one day multifocal punctate lesions were also seen away from the needle track that disappeared by five days. These lesions may represent mild, reversible astrocyte injury. A recent paper reported diffuse punctate lesions in ~60% of seropositive rats injected with IL-1β, but without lesions around the needle track [18]. In that study the level of NMO patient-derived IgG in rat serum was not measured, though it was likely to be quite low, as a single intraperitoneal injection of 10 mg of NMO patient-derived IgG in rat is much lower than 75 mg used in our study, which was chosen to produce serum cytotoxicity similar to that in human NMO. We speculate that the substantially lower serum NMO-IgG concentration in the study of Kitic et al. [18] was responsible for absence of robust NMO pathology.

## Conclusions

A robust, passive-transfer model of NMO was established involving intracerebral needle stab injury in rats made seropositive by IP administration of NMO-IgG. The model does not require administration of complement or pro-inflammatory factors, or pre-existing inflammation. The model should be useful for further evaluation of NMO pathogenesis mechanisms and for evaluation of NMO therapeutics targeting circulating NMO-IgG, NMO-IgG binding to AQP4, complement, and inflammatory cells and factors.

## Abbreviations

ADCC: Antibody-dependent cellular cytotoxicity; AQP4: Aquaporin-4; CDC: Complement-dependent cytotoxicity; EAE: Experimental autoimmune encephalomyelitis; GFAP: Glial fibrillary acidic protein; IP: Intraperitoneal; MBP: Myelin basic protein; NMO: Neuromyelitis optica; NMO-IgG: Neuromyelitis optica immunoglobulin G antibody.

## Competing interests

The authors declare that they have no competing interests.

## Authors’ contributions

NA: carried out experimental work and wrote manuscript draft; ASV: designed experiments and edited manuscript. Both authors read and approved the final manuscript.
